# LncRNA LINC00473 is involved in the progression of invasive pituitary adenoma by upregulating KMT5A via ceRNA-mediated miR-502-3p evasion

**DOI:** 10.1038/s41419-021-03861-y

**Published:** 2021-06-05

**Authors:** Junjun Li, Yuan Qian, Chao Zhang, Wei Wang, Yisheng Qiao, Hao Song, Liyan Li, Jiazhi Guo, Di Lu, Xingli Deng

**Affiliations:** 1 Institute of Neuroscience, Kunming Medical University, Kunming, China; 2 Department of Neurosurgery, 1st Affiliated Hospital of Kunming Medical University, Kunming, China; 3 Yunnan Key Laboratory of Laboratory Medicine, Yunnan Engineering Technology Center of Digestive disease, 1st Affiliated Hospital of Kunming Medical University, Kunming, China; 4 Genetic Diagnosis Center, Women and Children Hospital, Kunming, China; 5 Department of Neurosurgery, Affiliated Hospital of Hubei University of Medicine, Xiangyang, China; 6 Department of Neurosurgery, The People’s Hospital of Chuxiong, Chuxiong, China; 7 Biomedical Engineering Research Center, Kunming Medical University, Kunming, China

**Keywords:** Prognostic markers, Pituitary diseases, Prognostic markers, Oncogenesis

## Abstract

Long noncoding RNAs (lncRNAs) and their crosstalks with other RNAs have been revealed to be closely related to tumorigenesis and development, but their role in invasive pituitary adenoma (IPA) remains largely unclear. In our study, LINC00473 was identified as the most upregulated lncRNA in IPA by whole transcriptome RNA sequencing (RNA-Seq). Further, its related signaling pathway LINC00473/miR-502-3p/KMT5A was obtained by constructing a competing endogenous RNA (ceRNA) regulatory network. Their expression in IPA and non-invasive pituitary adenoma (NIPA) tissues was verified by qRT-PCR. Then the effects and mechanisms of LINC00473 and its ceRNA network on the proliferation of pituitary adenoma (PA) cells were confirmed by gene overexpression or silencing techniques combined with CCK-8 assay, EdU staining, flow cytometry assay, and double luciferase reporter gene assay in PA cell lines AtT-20 and GT1-1 in vitro and in a xenograft model in vivo. LINC00473 is overexpressed in IPA and can promote PA cells proliferation. Mechanistically, overexpression of LINC00473 restricts miR-502-3p through the ceRNA mechanism, upregulates KMT5A expression, and promotes the expression of cyclin D1 and CDK2, which is conducive to the cell cycle process, thereby promoting the proliferation of PA cells, involving IPA progression.

## Introduction

PA is one of the most common primary brain tumor and account for ~16% of intracranial tumors^1^. Although PA is a benign tumor, about 30% of them exhibit invasive behaviors and actively invade surrounding tissues, which are called IPAs^2,3^. Because IPAs are usually massive, rapidly growing, and severe destruction of the surrounding structures, the incidence of serious surgical complications and recurrence rate of surgical resection of IPA is significantly higher than that of NIPA^4^. Therefore, investigations on the molecular pathogenesis of IPA are urgently needed to develop novel therapeutic strategies for this tumor.

LncRNAs are RNAs with a transcript length of > 200 nucleotides^5^, and microRNAs (miRNAs) are single-stranded RNAs with a length of about 22 nucleotides^6^. Both of them belong to noncoding RNAs (ncRNAs) and do not contain protein-coding sequences, but they play important regulatory roles in various cellular processes, including transcriptional regulation, RNA modification, chromosome remodeling, protein transport, etc^5,6^. Meanwhile, increasing evidence has shown that their mutation and dysregulation are closely related to tumor progression, recurrence, and resistance to treatment^7–9^. It has been confirmed in many cancers that lncRNAs play important roles as ceRNAs competitively binding to shared miRNAs^10^. Similar studies also have been reported in the benign brain tumor PA. Overexpression of lncRNA RPSAP52 is related to the development of PA, which acts as a ceRNA to competitively binding miR-15 and miR-16, thereby enhancing their target protein HMGA expression and promoting tumor cells growth^11^. However, the human genome encodes more than 10,000 lncRNAs, and only a few lncRNAs are currently characterized^12,13^. The biological function and molecular mechanisms of lncRNA in PA still remain largely unknown.

In order to investigate the role of various RNAs and their crosstalks in IPA, we performed RNA-Seq of IPA and NIPA to identify differentially expressed (DE) RNAs between them. Meanwhile, we constructed a ceRNA network (lncRNA-miRNA-mRNA) through bioinformatics analysis. The results showed that LINC00473 was the most significantly upregulated lncRNA in IPA, an intergenic lncRNA from chromosome 6q27 that has been found to be overexpressed as an oncogene in many cancers^14^. However, its role in IPA is still unknown. Our further research found that LINC00473 may function through the ceRNA network LINC00473/miR-502-3p/KMT5A. Previous studies have revealed that miR-502 plays a role as a tumor suppressor gene in many cancers^15^, while the overexpression of the methyltransferase KMT5A may be involved in cancer progression^16^. Therefore, in this study, we detected the expression of LINC00473, miR-502-3p, and KMT5A in human PA tissues and investigated their role in IPA.

## Materials and methods

### Tumor tissue samples

IPA (*n* = 20) and NIPA (*n* = 20) tissues were collected from the First Affiliated Hospital of Kunming Medical University. The clinical characteristics of the patients are shown in Table 1. The patients did not undergo any treatment before surgery and all samples were confirmed by pathological examination. The criteria for IPA include: Knosp classification of pituitary tumors was grade III–IV, and intraoperative findings of tumor invasion (invasion of the dura and/or bone) and pathological reports of Ki-67 ≥ 3%^17^. This study was approved by the ethics committee of the first affiliated hospital of Kunming Medical University and each subject’s written informed consent was obtained.

**Table 1:** The clinical characteristics of the patients.

		IPA	NIPA
		*n* = 20	*n* = 20
***Average age***			
(Years)		39.2	43.7
Gender	Female	9	12
	Male	11	8
Knosp	I	0	11
	II	0	9
	III	7	0
	IV	13	0
Ki-67	<1%	0	14
	1–3%	0	6
	3–5%	17	0
	>5%	3	0

### Cell lines and cell culture

The PA cell lines AtT-20 and GT1-1 were purchased from Saiqi Biological Engineering co., ltd. (Saiqi, Shanghai, China). Cells were cultured in DMEM High Glucose (Corning, NY, USA) with 10% FBS (BI, Kibbutz Beit Haemek, Israel), 100 U/mL penicillin, and 100 µg/mL streptomycin, and placed in 37 °C, 5% CO_2_ cell incubator.

### Total RNA isolation, library preparation, and sequencing

Total RNA was isolated from IPA and NIPA tissues using miRNeasy Mini Kit (QIAGEN, Dusseldorf, Germany). According to the manufacturer’s recommendations, RNA concentration and integrity were measured by Qubit^®^ RNA HS Assay Kit (Life Technologies, CA, USA) and RNA Nano 6000 Assay Kit (Agilent Technologies, CA, USA), and libraries were prepared using NEBNext^®^ Ultra™ Directional RNA Library Prep Kit for Illumina^®^ (NEB, MA, USA). The final libraries were purified by the Agencourt AMPure XP system (BECKMAN COULTER, CA, USA) and sequenced using the Illumina Hiseq 4000 (Illumina, CA, USA).

### RNA-Seq analysis

The quality of all raw sequencing data was evaluated using FastQC. The transcripts were combined using Cuffmerge software and transcripts with indeterminate strand orientation were removed. Next, the transcripts with exon number ≥ 2 and length > 200 bp were selected and screened by Cufflinks software. Finally, the coding potential was predicted by PhyloCSF, Pfam, CPC, and CNCI, and the intersection of transcripts with no coding potential in these software analysis results was taken as the final lncRNA library.

The raw small RNA (sRNA) reads were screened to remove the reads with incorrect joints and low quality. Next, the reads with a length between 18nt and 35nt were selected and mapped to the reference sequence using Bowtie2. The mapped mature/hairpin were aligned with the specified range of sequences in miRbase to obtain known miRNAs. The novel miRNAs were obtained by integrating the results of the miRNA prediction software miREvo and mirdeep2. The expression levels of the known and novel miRNAs were normalized using TPM to obtain the final miRNA library. Similarly, mRNA reads were aligned with UCSC human genome hg19 using Bowtie2 to obtain the final mRNA library.

The DE RNAs between IPA and NIPA were identified by Cuffdiff 2.0 with |fold change *|* >2, *P* value < 0.05, and false discovery rate < 0.05.

### qRT-PCR

The total RNA was extracted using TRNzol-A^+^ reagent (TIANGEN, Beijing, China) and reversed transcribed using GoldenstarTM RT6 cDNA Synthesis Kit (TsingKe, Beijing, China). qRT-PCR was performed with SYBR Green І reagent (TsingKe, Beijing, China) and Bio-Rad CFX96 PCR System (BIO-RAD, CA, USA) with GAPDH as a reference gene. The primers were listed as follows:

LINC00473: forward, 5′-TCATTTCCCTACCTGCTCCT-3′; reverse, 5′-CAGTGTCTGCACATCGCTAAT-3′; miR-502-3p: forward, 5’-GTGCAGGGTCCGAGGT-3’; reverse, 5’-CGAATGCACCTGGGCAAG-3’; KMT5A: forward, 5’-GAACGTTCCCTCACTCCACC-3’; reverse, 5’-AAAGCCCATCAAGGGCAAAC-3’; GAPDH: forward, 5’-AGCCACATCGCTCAGACAC-3’; reverse, 5’-GAGGCATTGCTGATGATCTTG-3’.

### Cell transfection

Overexpression or knockdown of LINC00473/KMT5A by transfection of specific pcDNA 3.1 (+) or small interfering RNA (siRNA), overexpression, or inhibition of miR-502-3p by transfection of miR-502-3p-mimic or inhibitor, all reagents were purchased from Guangzhou RIOBIO Biotechnology Co., Ltd (RIOBIO, Guangzhou, China). Cells were transfected with Lipofectamine 2000 (Invitrogen, CA, USA), and the transfection efficiency was evaluated by qRT-PCR.

### Cell counting Kit-8 (CCK-8) assay

AtT-20 and GT1-1 cells were seeded in a 96-well plate, 10 μl of CCK-8 reagent (Dojindo, Kumamoto, Japan) was added to each well and incubated 1.5 h in the cell culture incubator. The absorbance was measured at 450 nm using SpectraMax M5 (Molecular Devices, CA, USA), and the proliferation curve was drawn based on the measurement results.

### EdU staining

AtT-20 and GT1-1 cells were seeded in a 24-well plate, 300 μL of EdU (50 μmol/L) reagent (RIOBIO, Guangzhou, China) was added to each well and incubated at 37 °C for 4 h. Next, added 200 μL of PBS containing 4% paraformaldehyde to the wells, incubated for 30 min at room temperature, permeabilized cells with Triton X-100, and added 100 μL of 1X Apollo^®^ 488 staining reaction solution to each well for 30 min. Wash with PBS after each step. Finally, observed under a fluorescence microscope (OLYMPUS, Tokyo, Japan).

### Flow cytometer assay

AtT-20 and GT1-1 cells were seeded in a 6-well plate overnight. The cells were digested with trypsin and washed twice with PBS after centrifugation. The cells resuspended in binding buffer and added Annexin V-FITC and PI (Solarbio, Beijing, China) staining solution incubate for 10 min at room temperature in dark, and then analyze the cell cycle distribution by flow cytometry (BD, Biosciences, USA).

### Dual luciferase reporter gene assay

293 T cells were co-transfected with psiCHECK2-LINC00473-wild or mut, psiCHECK2-KMT5A-wild or mut, and NC-mimic or miR-502-3p-mimic, respectively. 48 h after transfection, luciferase activity was measured using a dual luciferase reporter assay system (Promega, WI, USA), and Renilla luciferase activity was used for standardization.

### Western blot analysis

The total proteins were extracted with RIPA lysis buffer containing PMSF (Solarbio, Beijing, China) and quantified with BCA protein assay kit (Beyotime, Shanghai, China). The proteins were separated by SDS-PAGE (LEAGENE, Beijing, China) and transferred to the PVDF membranes (Millipore, MA, USA). The membranes were washed with TBST (Solarbio, Beijing, China) and sealed with skim milk, the primary antibodies were added and incubated overnight at 4 °C. The primary antibodies include: anti-cyclin D1 (Abcam, ab16663, Cambridge, UK), anti-CDK2 (Abcam, ab245310, Cambridge, UK), anti-KMT5A (Abcam, ab111691, Cambridge, UK), and anti-β-actin (Abcam, ab8227, Cambridge, UK). Next, the membranes were incubated with the corresponding secondary antibody (Abcam, ab205718, Cambridge, UK) for 90 min at room temperature. Proteins were detected using super ECL-Plus reagent (Millipore, MA, USA) and quantified using Image J software.

### Xenograft model

About 4–5 weeks old athymic female nude mice (nu/nu) were purchased from the Experimental Animal Center of Kunming Medical University and kept under the condition of specific pathogen-free. Then randomly divided into three groups, 200 μL of PBS containing 5 × 10^7^ normal, LINC00473 overexpressing, or LINC00473 knockdown AtT-20 cells were independently injected subcutaneously into the right hind back. Three per group. The tumor volumes of the mice were measured every week after implantation, and the tumor volume was calculated by length × width^2^ × 0.5. All mice were sacrificed 6 weeks later, tumors were harvested, then photographed, and subjected to subsequent studies. The GT1-1 cell strain was repeated in the same manner. All animal studies were approved by the Animal Experimental Committee of the First Affiliated Hospital of Kunming Medical University.

### Immunohistochemistry

The harvested xenograft model nude mice tumors were fixed and embedded in paraffin. The tumor tissues were sliced, deparaffinized, and hydrated, then the endogenous peroxidase and non-specific proteins were blocked, and the primary antibody was added and incubated at 4 °C Overnight. The primary antibodies include anti-ki-67 (Abcam, ab231172, Cambridge, UK), anti-KMT5A (Abcam, ab111691, Cambridge, UK). Next, the slices are washed with PBS and incubated with secondary antibody (Abcam, ab205718, Cambridge, UK) at 37 °C for 30 min, developed with DAB, counterstained with hematoxylin, dehydrated, and observed under an optical microscope.

### Statistical analysis

The data were statistically analyzed using SPSS 22.0, and the results were expressed as mean ± SD. The comparison between the two groups was performed by Student’s *t*-test, and the comparison between multiple groups was performed by one-way analysis of variance. *P* < 0.05 was considered statistically significant. All data are verified by at least three independent experiments.

## Results

### Profile of RNA expression in PA tissue

**Fig. 1:**
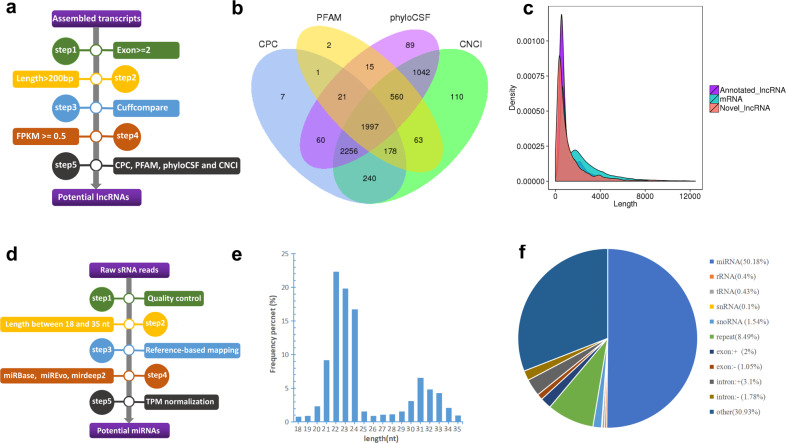
Profile of RNA expression in PA tissue. **a** Workflow of lncRNA analysis and screening. **b** Venn diagram showing coding potential screening results. **c** Comparison of the length of lncRNA and mRNA. **d** Workflow of sRNA analysis and screening. **e** Length distribution of sRNA fragments in IPA and NIPA libraries. **f** Classification and proportion of all sRNAs.

To identify possible transcripts in human IPA, we used total RNA from four IPAs and three NIPAs samples to construct RNA libraries and acquired 81–97 million uniquely mapped clean reads from each of the IPA and NIPA libraries, respectively. Next, the lncRNA was identified according to the workflow steps shown in Fig. 1a. After screening for the coding potential, a total of 1997 candidate lncRNAs were identified (Fig. 1b), and 19,158 candidate mRNAs were identified in the RNA libraries. Analysis of lncRNA characterization revealed that the average length of lncRNAs was 1346nt, which was shorter than the length of mRNA (2206nt) (Fig. 1c).

Similarly, the sRNA libraries were constructed according to the workflow steps shown in Fig. 1d. After filtering the raw reads, we obtained a total of 10–15 million and 10–14 million clean reads from each of the IPA and NIPA libraries, respectively. The length of human miRNAs is usually 21–25nt. In this study, the proportion of reads with the length of 21–25nt in total sRNA was 69.57% (Fig. 1e). The classification (including known miRNA, rRNA, tRNA, snRNA, snoRNA, repeat RNA, gene, and novel miRNA) and annotation of all sRNAs obtained from PA libraries are shown in Fig. 1f, and the proportion of miRNA in the total sRNA was 50.18%.

### Characterization of LINC00473 and its corresponding ceRNA network (lncRNA-miRNA-mRNA) in IPA

**Fig. 2:**
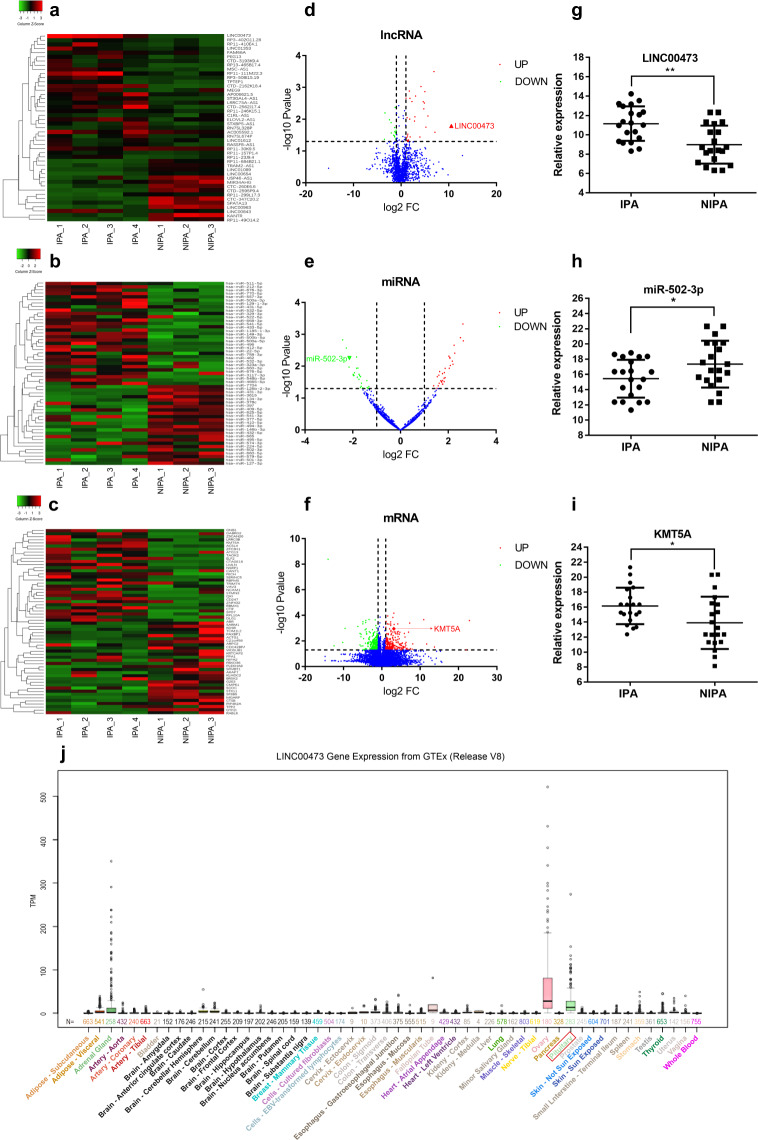
Characterization of LINC00473 and its corresponding ceRNA network (lncRNA-miRNA-mRNA) in IPA. **a**–**c** Heat map showing the DE lncRNAs, miRNAs, and mRNAs between IPA and NIPA. **d**–**f** Volcano plot showing DE lncRNAs, miRNAs, and mRNAs between IPA and NIPA, with red dots indicating upregulation and green dots indicating downregulation. The red positive triangle in (**d**) is displayed as LINC00473, the green inverted triangle in (**e**) is displayed as miR-502-3p, and the red diamond in (**f**) is displayed as KMT5A mRNA. **g**–**i** qRT-PCR analysis of the expression of LINC00473, miR-502-3p, and KMT5A mRNA in IPA (*n* = 20) and NIPA(*n* = 20) tissues. **j** LINC00473 gene expression from GTEx, the red box shows the pituitary. *P < 0.05, **P < 0.01, data represent the mean ± SD.

Analysis of the corresponding RNA libraries of IPA and NIPA identified a total of 45 lncRNAs, 54 miRNAs and 533 mRNAs were significantly DE with |fold change *|* >2 and *P* < 0.05 (Fig. 2a–f). In IPA, LINC00473 was the most significantly upregulated lncRNA (Fig. 2d). To construct a ceRNA network related to LINC00473, we used a bioinformatics database (Starbase 2.0, http://starbase.sysu.edu.cn/) to predict miRNAs that have potential interactions with LINC00473. We found that LINC00473 has binding sites with many miRNAs, among which miR-502-3p is one of the miRNAs that is significantly down-regulated in IPA (Fig. 2e). Meanwhile, according to previous research reports, the target gene of miR-502-3p is KMT5A, which is one of the mRNAs significantly upregulated in IPA (Fig. 2f). Next, we detected the expression levels of LINC00473, miR-502-3p, and KMT5A mRNA in IPA (*n* = 20) and NIPA (*n* = 20) by qRT-PCR to verify the RNA-Seq results (Fig. 2g–i, *P* < 0.05). Finally, we obtained the potential ceRNA network of LINC00473 as LINC00473/miR-502-3p/KMT5A. In addition, the Genotype Tissue Expression (GTEx) (https://www.gtexportal.org) data show that LINC00473 has a higher expression level in normal pituitary tissues (Fig. 2j).

### LINC00473 promotes PA cells proliferation in vitro

**Fig. 3:**
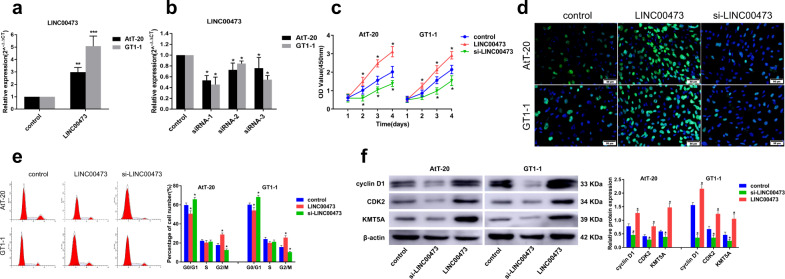
LINC00473 promotes PA cells proliferation in vitro. qRT-PCR detection of the plasmid (**a**) and siRNA (**b**) transfection efficiency in PA cells AtT-20 and GT1-1. **c** CCK-8 detection of PA cells viability after overexpression or knockdown of LINC00473. **d** Cell proliferation was determined by EdU staining. **e** Cell cycle was analyzed by flow cytometry. **f** Western blot analysis of cyclin D1, CDK2, and KMT5A expression. *P < 0.05, **P < 0.01, ***P < 0.001, data represent the mean ± SD.

The results of isolated PA tissues indicate that LINC00473 is closely related to the invasion of PA (Fig. 2). To further verify this result, we transfected PA cells AtT-20 and GT1-1 by plasmid or siRNA to overexpress or knockdown LINC00473, and examined their effects on cell proliferation. First, we tested the transfection efficiency by qRT-PCR, and the results showed that the PA cells overexpressed LINC00473 after plasmid transfection (Fig. 3a). At the same time, we designed three kinds of LINC00473 siRNA and transfected into PA cells, and found that siRNA-1 inhibited the expression of LINC00473 most significantly (Fig. 3b). Next, we detected the influence of different conditions on the proliferation of PA cells. The results of the CCK8 assay showed that the activity of PA cells increased significantly after overexpression of LINC00473, but decreased significantly after knockdown (Fig. 3c). EdU staining analysis showed that the proliferation of PA cells was significantly promoted by overexpression of LINC00473, but significantly inhibited by knockdown (Fig. 3d). Meanwhile, overexpression of LINC00473 facilitated cell transition from G1 to S, while knockdown of LINC00473 led to cell cycle arrest (Fig. 3e). Cyclin D1 and CDK2 are key regulatory proteins for the transition of the cell cycle from G1 to S. We examined their expression by Western blot. The results showed that the expression of cyclin D1, CDK2, and KMT5A can be significantly promoted when LINC00473 was overexpressed, while the result was opposite when LINC00473 was knocked down (Fig. 3f). These results indicate that LINC00473 may promote the proliferation of PA cells by regulating the cell cycle.

### LINC00473 counteract the inhibitory effect of miR-502-3p on PA cells proliferation

**Fig. 4:**
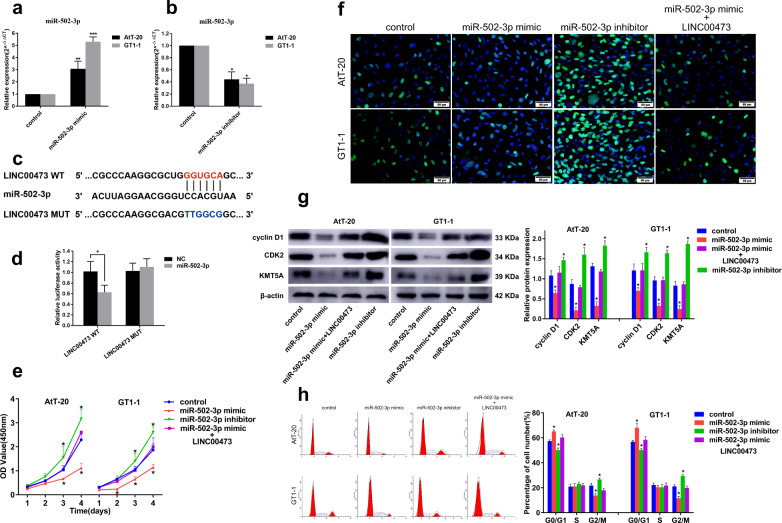
LINC00473 counteract the inhibitory effect of miR-502-3p on PA cells proliferation. qRT-PCR detection of miR-502-3p mimic (**a**) and inhibitor (**b**) transfection efficiency in PA cells AtT-20 and GT1-1. **c** Prediction of binding sites for LINC00473 and miR-502-3p, wide type and mutant type of LINC00473 were designed for luciferase reporter assay. **d** Dual luciferase reporter gene assay was used to detect the binding ability between LINC00473 and miR-502-3p. **e** CCK-8 detection of PA cells viability after transfection of miR-502-3p mimic, inhibitor or co-transfection of miR-502-3p mimic and pcDNA 3.1(+)-LINC00473. **f** Cell proliferation was determined by EdU staining. **g** Western blot analysis of cyclin D1, CDK2, and KMT5A expression. **h** Cell cycle was analyzed by flow cytometry. *P < 0.05, **P < 0.01, ***P < 0.001, data represent the mean ± SD.

To further verify the effect of miR-502-3p and its relationship with LINC00473 in PA, miR-502-3p mimic or inhibitor was transfected into PA cells AtT-20 and GT1-1 to overexpress or knockdown miR-502-3p, moreover, simultaneously overexpressed miR-502-3p and LINC00473, and tested for their effects on cell proliferation. First, we tested the transfection efficiency of miR-502-3p mimic and inhibitor by qRT-PCR (Fig. 4a, b). Next, we verified the existence of a functional interaction site between miR-502-3p and LINC00473 by dual luciferase reporter gene assay. The binding sites and corresponding mutation sites between LINC00473 and miR-502-3p are shown in Fig. 4c. The results showed that miR-502-3p mimic significantly reduced the luciferase activity of LINC00473-WT, but had no significant effect on LINC00473-MUT (Fig. 4d). Then we detected the effect of different conditions on proliferation of PA cells. The results of the CCK8 assay showed that miR-502-3p inhibitor increased the activity of PA cells, while its mimic inhibited the cells activity, and this inhibitory effect could be offset by overexpression of LINC00473 (Fig. 4e). EdU staining assay result (Fig. 4f) was consistent with CCK-8. MiR-502-3p inhibitor significantly promoted the proteins expression of cyclin D1, CDK2, and KMT5A, while miR-502-3p mimic inhibited their expression. In addition, simultaneous overexpression of miR-502-3p and LINC00473 showed no significant effect on the expression levels of these proteins (Fig. 4g). Flow cytometry analysis showed that miR-502-3p mimic led to cell cycle arrest in G1 to S, while miR-502-3p inhibitor facilitated cell transition from G1 to S. The effect of miR-502-3p mimic on the cell cycle can also be offset by overexpression of LINC00473 (Fig. 4h). These results indicate that LINC00473 may promote cell cycle progression through negative regulation of miR-502-3p.

### LINC00473/miR-502-3p affects proliferation of PA by targeting KMT5A

**Fig. 5:**
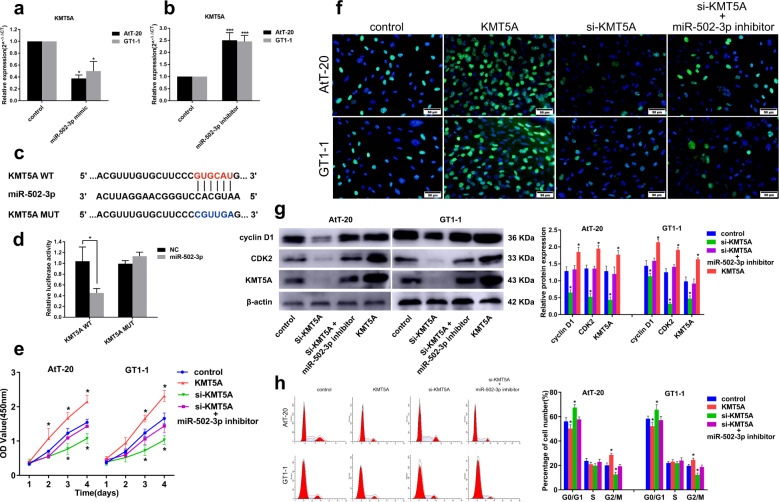
The role of KMT5A in promoting proliferation of PA cells was regulated by miR-502-3p. qRT-PCR detection of KMT5A mRNA expression in PA cells AtT-20 and GT1-1 after transfection of miR-502-3p mimic (**a**) and inhibitor (**b**). **c** Prediction of binding sites for KMT5A and miR-502-3p, wide type and mutant type of KMT5A were designed for luciferase reporter assay. **d** Dual luciferase reporter gene assay was used to detect the binding ability between KMT5A and miR-502-3p. **e** CCK-8 detection of PA cells viability after overexpression of KMT5A, knockdown of KMT5A, or knockdown of KMT5A while inhibition of miR-502-3p. **f** Cell proliferation was determined by EdU staining. **g** Western blot analysis of cyclin D1, CDK2, and KMT5A expression. **h** Cell cycle was analyzed by flow cytometry. *P < 0.05, **P < 0.01, ***P < 0.001, data represent the mean ± SD.

The crosstalk of LINC00473 and miR-502-3p can affect the proliferation of PA, but they all belong to ncRNAs and need to exert their biological functions by regulating their target mRNAs. We speculate that KMT5A may be their target gene, and we have further verified this. The results showed that the expression of KMT5A mRNA was negatively correlated with the expression of miR-502-3p in PA cells (Fig. 5a, b). Similarly, we verified the existence of a functional interaction site between KMT5A and miR-502-3p by dual luciferase reporter gene assay. The binding sites and corresponding mutation sites between KMT5A and miR-502-3p are shown in Fig. 5c. The results showed that miR-502-3p mimic significantly reduced the luciferase activity of KMT5A-WT but had no significant effect on KMT5A-MUT (Fig. 5d). The studies of the effect of KMT5A on the proliferation of PA cells shown that low expression of KMT5A significantly inhibited the cell viability (Fig. 5e) and proliferation (Fig. 5f), and decreased cell cycle-related protein expression (Fig. 5g), and led to cell cycle arrest (Fig. 5h), which could be counteracted by simultaneously knocking down miR-502-3p. These results indicate that the effect of LINC00473 in promoting the proliferation of PA cells may be exerted by inhibiting miR-502-3p to upregulate KMT5A.

### LINC00473 promotes the growth of PA in vivo

**Fig. 6:**
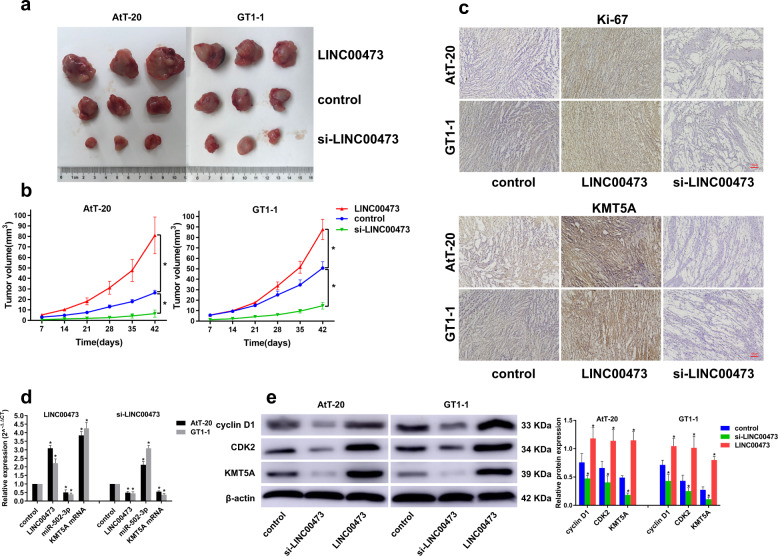
LINC00473 promotes the growth of PA in vivo. **a** Images of tumors formed in nude mice injected subcutaneously with PA cells (overexpression, control, or knockdown of LINC00473) were shown. **b** The volume of subcutaneous xenograft tumors. **c** Ki-67 and KMT5A immunohistochemical staining. **d** qRT-PCR was used to detect the expression of LINC00473, miR-502-3p, and KMT5A mRNA in tumor tissues after overexpression or knockdown of LINC00473. **e** Western blot analysis of cyclin D1, CDK2, and KMT5A expression in tumor tissues. *P < 0.05, **P < 0.01, data represent the mean ± SD.

The role of LINC00473 in promoting the proliferation of PA has been confirmed in isolated tumor tissues and in vitro PA cell lines. To further validate its effect on PA growth in vivo, we established xenograft models of PA cells that overexpressed or knocked down LINC00473. The results showed that overexpression of LINC00473 significantly promoted tumor growth (Fig. 6a), and the xenograft tumors had the larger volume (Fig. 6b) and the higher Ki-67 and KMT5A positive rate (Fig. 6c). Meanwhile, the expression level of LINC00473 also significantly affected the expression of miR-502-3p and KMT5A mRNA (Fig. 6d). The results of cell cycle related protein expression were consistent with the results in vitro (Fig. 6e).

## Discussion

The common feature of IPAs is that cells proliferate vigorously, and tumor proliferative capacity is the most important indicator for measuring invasiveness^18^. Therefore, identifying the reason of tumor cell proliferation is the key to elucidating the pathogenesis of IPA. Multiple studies have found that the ncRNAs are frequently dysregulated in tumors and their complex interactions with mRNA may be involved in tumor occurrence and development^7,8^. Among the many ncRNAs, studies have confirmed that miRNA can regulate gene expression by degrading target mRNA or inhibiting the translation of target mRNA, while other ncRNAs may act as ceRNAs or miRNA sponges and perform biological functions by competitively binding shared miRNA^9^.

Many RNA crosstalks identified so far have been involved in human tumors. For instance, lncRNA-ATB can induce epithelial-mesenchymal transition (EMT) to promote hepatocellular carcinoma (HCC) invasion by competitively binding miR-200 to upregulate ZEB1 and ZEB2. In addition, lncRNA-ATB can also promote the colonization of HCC cells at metastatic sites by binding to IL-11 mRNA, inducing IL-11 autocrine and activating STAT3 signal transduction^19^. In glioma, lncRNA NEAT1 is upregulated, which can promotes tumor progression by inhibiting miR-132 to promote SOX2 expression^20^. In colon cancer, miR-145 can inhibit the proliferation and differentiation of colon cancer stem cells, but lncRNA CCAT2 can block this effect by preventing the maturation of pre-miR-145 to miR-145^21^.

These findings indicate that the dysregulation of various RNAs and the crosstalks between them are closely related to the proliferation and invasion of tumor cells. Therefore, we performed RNA-seq of IPA and NIPA tissues to analyze the DE RNAs between them. In our study, LINC00473 was identified as the most significantly upregulated lncRNA in IPA, and through bioinformatics analysis combined with previous research reports, the related ceRNA network was constructed as LINC00473/miR-502-3p/KMT5A.

Until now, numerous studies have found that LINC00473 plays an oncogene role in multiple tumors, including glioma^22,23^, Wilms tumor^24^, cervical cancer^25^, breast cancer^26–28^, esophageal squamous cell carcinoma^29,30^, lung cancer^31^, hepatocellular carcinoma^32–34^, cholangiocarcinoma^35^, gastric cancer^36^, colorectal cancer^37^, pancreatic cancer^38^, and mucoepidermoid carcinoma^39^, etc. It is closely related to tumor proliferation, migration, invasion, and chemo/radio-resistance, and its high expression indicates a poor prognosis. We have demonstrated that upregulate LINC00473 can also significantly promote the proliferation of PA cells in vitro and in vivo. Mechanistically, the most recognized is that LINC00473 acts as a ceRNA competitive binding shared miRNA to affect the expression of target genes, thereby regulating cell cycle, apoptosis, signal transduction or EMT, and involving in tumor occurrence and development. In different tumors, LINC00473 may play a regulatory role by crosstalk with different miRNAs. Previously reported LINC00473 competitively binding and regulated miRNAs include miR-34a^25^, miR-198^26^, miR-497^27,29^, miR-374a-5p^30^, miR-195^32,38^, miR-29a-3p^34^, miR-506^35^, and miR-15a^37^, etc. In our study, miR-502-3p was identified as the target miRNA of LINC00473, and its expression is negatively correlated with LINC00473 and tumor invasiveness in PA. Furthermore, the interaction between them was confirmed by dual luciferase reporter gene assay, and LINC00473 can negatively regulate the expression of miR-502-3p in PA cells. Their role in PA cells is that the overexpression of LINC00473 promotes cells proliferation, while the overexpression of miR-502-3p inhibits cells proliferation. The inhibitory effect of miR-502-3p can be neutralized by LINC00473. The role of LINC00473 in promoting the proliferation of PA was also validated in xenograft models. These results indicate that LINC00473 may inhibit miR-502-3p through ceRNA mechanism to promote PA cells proliferation.

Previous studies have found that miR-502 can inhibit tumor progression in many tumors. PCNA and MMP are closely related to the occurrence and metastasis of gastric cancer. MiR-502 can inhibit their expression by down-regulating NRAS and inhibiting the activation of NRAS/MEK1/ERK1/2 signaling pathway^40^. MiR-502 also inhibit the proliferation and metastasis of hepatoma cells by inhibiting Snail-mediated EMT^41^ or by modulating cell cycle through suppressing KMT5A^15^ and phosphoinositide 3-kinase catalytic subunit gamma^42^. Certainly, among the numerous target genes regulated by miR-502, the most common and thorough studied gene is the KMT5A, which has a binding site of miR-502 in its 3’untranslated region (UTR). MiR-502 can bind to it and then regulate the expression of the protein encoded by KMT5A to exert the function of a tumor suppressor^43^. In our study, the target mRNA for miR-502-3p is KMT5A.

KMT5A (also known as SET8, SETD8, or PR-SET7) is histone H4 Lys20 (H4K20me1) specific histone methyltransferase containing set domain, which involves many essential cellular processes through methylation of H4K20, including cell cycle regulation, maintenance of gene stability, chromosome remodeling, and transcriptional regulation^44^. In addition, KMT5A can also monomethylate non-histone substrates including PCNA and p53^16,45^. Under normal physiological conditions, the level of H4K20me1 and KMT5A changes dynamically in different stages of the cell cycle, which ensures normal cell cycle progression^46^. Recent studies have found that the single nucleotide polymorphism (SNP) of the miR-502 binding site in KMT5A 3’UTR is closely related to the occurrence and development of many tumors^43,47^, and the patients with CC genotype SNP have a better prognosis, and this genotype is considered to have a stronger affinity with miR-502, while downregulating KMT5A expression^48^. Similarly, some studies have found that KMT5A is abnormally overexpressed in many tumors and is positively correlated with the prognosis of patients, which may promote tumor cells proliferation by enhancing aerobic glycolysis^49^ or lipid metabolism^50^, activating androgen-induced cell proliferation^51^, or inhibiting Numb-p53-mediated cell apoptosis^45^. Meanwhile, it can also promote tumor metastasis by interacting with the EMT-related factor TWIST or ZEB1^52,53^.

In this study, we found that KMT5A mRNA was overexpressed in IPA tissues and confirmed the presence of a binding site between KMT5A and miR-502-3p. The expression level of KMT5A in PA cells is positively correlated with LINC00473 and negatively correlated with miR-502-3p. Simultaneously, LINC00473 can counteract the inhibitory effect of miR-502-3p on KMT5A. Cyclin D1 and CDK2 are key proteins required for cells to enter the S phase from the G1 phase and are essential for normal cell growth and development^54^, but their high expression in many cancers is found to be involved in tumor progression^55,56^. We found that upregulation of KMT5A increased the expression of cyclin D1 and CDK2 in PA cells, accompanied by a decrease in G1 phase cells, an increase in S phase cells, and enhanced cells viability, suggesting that KMT5A may promote PA cells proliferation by facilitating cell cycle progression.

**Fig. 7:**
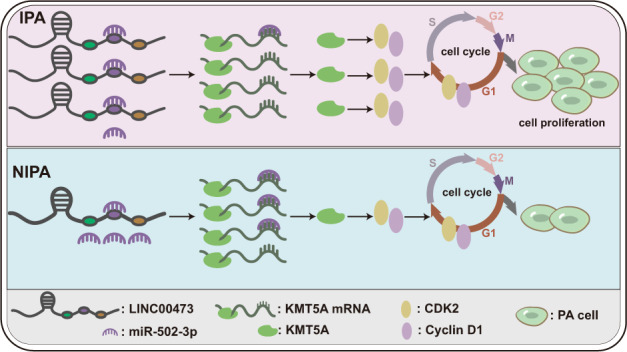
Hypothesis diagram of the role and mechanism of LINC00473/miR-502-3p/KMT5A axis in IPA. The overexpression of LINC00473 in IPA upregulates KMT5A by inhibiting miR-502-3p, thereby upregulating the expression of cyclin D1 and CDK2, facilitating cell cycle progression and promoting cell proliferation.

In summary, we uncovered that overexpression of LINC00473 in IPA upregulates KMT5A by ceRNA-mediated miR-502-3p evasion, and the upregulation of KMT5A increases expression of cyclin D1 and CDK2, the key proteins of the cell cycle, which favors cell cycle progression and promote cell proliferation, leading to tumor proliferation and invasion (Fig. 7).
